# Retraction: Opposing signaling pathways regulate morphology in response to temperature in the fungal pathogen *Histoplasma capsulatum*

**DOI:** 10.1371/journal.pbio.3002060

**Published:** 2023-03-21

**Authors:** Lauren Rodriguez, Mark Voorhies, Sarah Gilmore, Sinem Beyhan, Anthony Myint, Anita Sil

After this article [1] was published, the authors conducted further genotypic and phenotypic analyses that revealed that the morphological phenotype studied in the article was not due to an Msb2 mutation but rather to an unlinked chromosome alteration. The nature of the chromosomal alteration is still under investigation. Given this new information, statements in [1] attributing outcomes to Msb2 or an *msb2* mutation are not supported, nor are the article’s main conclusions about Msb2 functions and temperature-dependent antagonism between the Ryp and Msb2 pathways.

In addition, the authors have been unable to reproduce the finding that *STU1* gene is partially required for hyphae formation at room temperature. The authors hypothesize that the phenotype may have been due to the variable nature of the filamentation assay.

In light of these issues, the authors retract this article.

All authors agreed with the retraction. We apologize that these issues were not identified before the article was published.
